# Peptide YY (PYY) Is Associated with Cardiovascular Risk in Patients with Acute Myocardial Infarction

**DOI:** 10.3390/jcm9123952

**Published:** 2020-12-06

**Authors:** Elias Haj-Yehia, Robert Werner Mertens, Florian Kahles, Marcia Viviane Rückbeil, Matthias Rau, Julia Moellmann, Moritz Biener, Mohammad Almalla, Jörg Schroeder, Evangelos Giannitsis, Hugo Albert Katus, Nikolaus Marx, Michael Lehrke

**Affiliations:** 1 Department of Internal Medicine I-Cardiology, University Hospital Aachen, Pauwelsstraße 30, 52074 Aachen, Germany; elias.haj.yehia@gmail.com (E.H.-Y.); romertens@ukaachen.de (R.W.M.); florian.kahles@me.com (F.K.); mrau@ukaachen.de (M.R.); jmoellmann@ukaachen.de (J.M.); malmalla@ukaachen.de (M.A.); jschroeder@ukaachen.de (J.S.); nmarx@ukaachen.de (N.M.); 2 Department of Medical Statistics, University Hospital Aachen, Pauwelsstraße 19, 52074 Aachen, Germany; mrueckbeil@ukaachen.de; 3 Department of Cardiology, Angiology, and Pneumology, Heidelberg University Hospital, Im Neuenheimer Feld 410, 69120 Heidelberg, Germany; Moritz.Biener@med.uni-heidelberg.de (M.B.); Evangelos.Giannitsis@med.uni-heidelberg.de (E.G.); hugo.katus@med.uni-heidelberg.de (H.A.K.)

**Keywords:** PYY, gut hormone, cardiovascular risk, mortality, myocardial infarction

## Abstract

Aims: Recent studies have found circulating concentrations of the gastrointestinal hormone GLP-1 to be an excellent predictor of cardiovascular risk in patients with myocardial infarction. This illustrates a yet not appreciated crosstalk between the gastrointestinal and cardiovascular systems, which requires further investigation. The gut-derived hormone Peptide YY (PYY) is secreted from the same intestinal L-cells as GLP-1. Relevance of PYY in the context of cardiovascular disease has not been explored. In this study, we aimed to investigate PYY serum concentrations in patients with acute myocardial infarction and to evaluate their association with cardiovascular events. Material and Methods: PYY levels were assessed in 834 patients presenting with acute myocardial infarction (553 Non-ST-Elevation Myocardial Infarction (NSTEMI) and 281 ST-Elevation Myocardial Infarction (STEMI)) at the time of hospital admission. The composite outcomes of first occurrence of cardiovascular death, nonfatal myocardial infarction, nonfatal stroke (3-P-MACE), and all-cause mortality were assessed with a median follow-up of 338 days. Results: PYY levels were significantly associated with age and cardiovascular risk factors, including hypertension, diabetes, and kidney function in addition to biomarkers of heart failure (NT-pro BNP) and inflammation (hs-CRP). Further, PYY was significantly associated with 3-P-MACE (HR: 1.7; 95% CI: 1–2.97; *p* = 0.0495) and all-cause mortality (HR: 2.69; 95% CI: 1.61–4.47; *p* = 0.0001) by univariable Cox regression analyses, which was however lost after adjusting for multiple confounders. Conclusions: PYY levels are associated with parameters of cardiovascular risk as well as cardiovascular events and mortality in patients presenting with acute myocardial infarction. However, this significant association is lost after adjustment for further confounders.

## 1. Introduction

Patients presenting with acute myocardial infarction are heterogeneous in clinical presentation and cardiovascular risk. While patients with ST-Elevation Myocardial Infarction (STEMI) require immediate revascularization, a more delayed intervention is acceptable for patients with Non-ST-Elevation Myocardial Infarction (NSTEMI) [1,2]. Decision making and optimal timing for coronary intervention of NSTEMI patients are facilitated by different risk scores, including the Global Registry of Acute Coronary Events (GRACE) and the Thrombolysis in Myocardial Infarction (TIMI) risk score [2]. Coronary revascularization improves prognosis of patients with acute myocardial infarction [3]. However, a residual risk remains. This is attributable to prevailing disease conditions and different cardiovascular risk factors. Understanding the mechanisms relevant for disease progression and prognosis is of major relevance for the implementation of existing and future therapies in patients with acute myocardial infarction.

Recent studies have identified gastrointestinal hormones as modulators of cardiovascular risk. The gut-derived peptide GLP-1 was found to be secreted in response to acute myocardial infarction and to independently predict adverse outcome in this patient population [4,5]. Importantly, GLP-1 receptor agonists reduced cardiovascular events in high-risk patients with diabetes [6,7,8,9].

Little knowledge exists about other gastrointestinal hormones and their relevance for cardiovascular disease. Among these, Peptide YY (PYY) is a 36 amino acid hormone released from the same enteroendocrine L-cells as GLP-1 [10,11]. PYY(1–36) is rapidly cleaved by dipeptidyl peptidase enzyme 4 (DPP-4) to create PYY(3–36) as the major circulating form of the peptide [12]. PYY(3–36) and PYY(3–36) both bind to the G-protein coupled receptors Y1 and Y2 with PYY(3–36) more potently activating the Y2 receptor [13,14]. PYY is known to downregulate gastrointestinal motility [15], cause intestinal vasoconstriction [16], and reduce appetite and body weight while improving glucose metabolism [17]. Interaction of PYY with the cardiovascular system has not been explored. In this study, we investigated the association of serum PYY concentrations with parameters of cardiovascular risk and mortality in patients presenting with acute myocardial infarction.

## 2. Methods

### 2.1. Study Population and Follow-Up

A total of 834 patients (mean age ± standard deviation (SD) = 66.9 ± 12.7 years; men 73.2%) were recruited after hospital admission at the University Hospital Heidelberg between 2006 and 2010. They presented with either STEMI (35%) or NSTEMI (65%). Refusal to provide written informed consent was the only exclusion criterion. The attending cardiologist was responsible for patient risk stratification, treatment, and management decisions. If a hospital admission to another hospital occurred for cardiovascular reasons, hospital dismissal reports were obtained and checked for a diagnosis of a cardiovascular event or death. Patients who were lost to follow-up were treated as censored observations in the Cox regression model. Hospital records, questionnaires, phone calls, and death certificates were used for the study follow-up. The study conformed to the Declaration of Helsinki. The research protocol was permitted by the locally assigned ethics committee, and written informed consent was afforded to all patients. At the moment of hospital admission, the GRACE score was calculated using the respective values of eight variables (age, heart rate, systolic blood pressure, serum creatinine concentration, Killip class, cardiac arrest, presence of ST-segment deviation, and elevated cardiac enzymes/markers) by using the GRACE risk calculator [18]. 

### 2.2. Laboratory Parameters

All blood samples were obtained by venipuncture at the time of admission in the chest pain unit (CPU) before initiation of medical treatment and angiography. Serum samples were stored at −80 °C. High-sensitivity Troponin T was determined in all patients by the fourth-generation Troponin T assay (Roche Diagnostics, Basel, Switzerland) until 2008, and after that, the COBAS E411 platform (Roche Diagnostics) was used for measurement. As a cutoff for indication of myocardial injury of 0.03 ng/mL was considered in all patients. *N*-terminal pro-B-type natriuretic peptide (NT-proBNP) levels were measured by an immunoassay on an Elecsys 2010 instrument (Roche Diagnostics). Total PYY levels were determined using a commercial ELISA kit (Millipore; #EZHPYYT66K, Burlington, MA, USA), which detects PYY(1–36) and PYY(3–36). The inter-assay variability of control serum run on each plate was 12.04%; the intra-assay variability was 8.38%. Assessment of the diagnosis and declaration of the type of myocardial infarction was performed by two independent cardiologists under inclusion of all available clinical data as well as angiography and imaging (echocardiography and magnetic resonance imaging if existing). This study was based on a retrospective analysis from frozen serum samples continuously stored at −80 °C. Additional clinical characteristics and parameters were evaluated retrospectively from the routine clinical documentation system.

### 2.3. Statistical Analysis

Continuous data are presented as mean and ±SD or as median with lower and upper quartile (Q1–Q3) in cases of skewed data. The skewness of characteristics was examined using boxplots and histograms. Categorical outcomes are given as absolute and relative frequencies (%).

The association between baseline characteristics and PYY tertiles (low: PYY ≤ 114 pg/mL, medium: PYY between 114 and 168 pg/mL, and high: PYY > 168 pg/mL) was assessed using the Cochran–Armitage test for nominal characteristics and the Spearman correlation coefficient *ρ* in the case of continuous characteristics. Kaplan–Meier cumulative event curves showed the all-cause mortality in dependence of PYY tertiles. Median follow-up times were computed separately for the composite outcome (3-P-MACE) and all-cause mortality by the reverse Kaplan–Meier method. The association between logarithmized PYY levels (and PYY tertiles) and both survival outcomes was examined using a univariable Cox regression model. In addition, multiple multivariable Cox regression models were computed to show the adjusted association of logarithmized PYY levels and all-cause mortality. Skewed data were logarithmically transformed to improve model stability. The proportional hazards assumptions were checked graphically using Schoenfeld residuals.

The significance level was set at *p* < 0.05. As this was an exploratory analysis, no further adjustments were made for multiple testing. All statistical analyses were performed using SAS software version 9.4 (SAS Institute, Cary, NC, USA) and R version 3.5.1 [19].

## 3. Results

### Baseline Characteristics

Total PYY serum concentrations were assessed at time of hospital admission in patients presenting with acute myocardial infarction. Clinical and laboratory baseline characteristics of the study population according to PYY tertiles are presented in Table 1a,b. PYY levels were significantly associated with age and cardiovascular risk factors, including hypertension and diabetes, but not smoking or hypercholesterolemia. In addition, PYY levels were associated with kidney function (GFR CKD-EPI), parameters of inflammation (hs-CRP), cardiac dysfunction (NT-proBNP), and muscular necrosis (CK although not hs-Troponin). Furthermore, PYY tertiles increased with increasing GRACE risk scores as an established risk calculator for patients with acute coronary syndrome (Table 1a). Consistent with PYY being associated to cardiovascular risk factors and diabetes, we found significantly more patients in the highest PYY tertile to receive blood pressure and diabetes medication in addition to lipid-lowering drugs and antiplatelet therapy (Table 1b).

**Figure 1:**
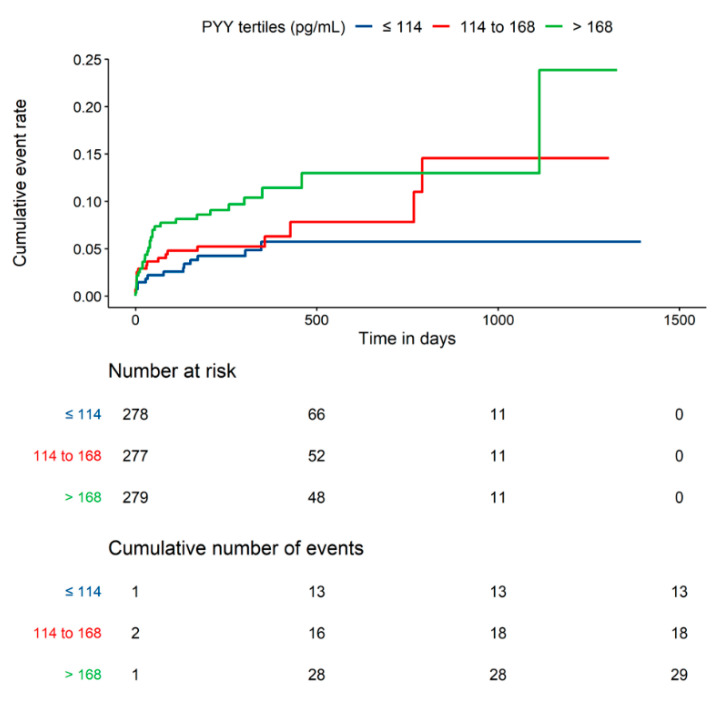
Kaplan–Meier cumulative event curves for all-cause mortality with patients separated by PYY tertiles.

A combined endpoint of the first occurrence of nonfatal myocardial infarction, nonfatal stroke, or cardiovascular death (3-P-MACE) was observed in 56 of the 834 patients (6.7%) (26 patients with nonfatal myocardial infarction, 3 patients with nonfatal stroke, and 27 patients with cardiovascular death). Death (all-cause mortality) occurred in 60 patients (7.2%) (Table 2). The median follow-up for 3-P MACE was 308 days, and 310 days for all-cause mortality. Using a univariable Cox regression model, logarithmized PYY serum levels were significantly associated with 3-P-MACE (HR: 1.7; 95% CI: 1.0–2.97; *p* = 0.0495). This was mainly attributable to a significant association of PYY with nonfatal myocardial infarction (HR: 2.54; 95% CI: 1.12–5.76; *p* = 0.0259) (Table 2). Furthermore, logarithmized PYY serum levels were significantly associated with all-cause mortality (HR: 2.69; 95% CI: 1.61–4.47; *p* = 0.0001) as depicted for PYY tertiles (Figure 1).

We considered several multivariable Cox regression analyses to adjust for different baseline characteristics. The association of PYY with total mortality remained significant after consideration of age and sex (HR: 2.26; 95% CI 1.33–3.83; *p* = 0.0024) (Table 3; multivariable Model 1). This association was, however, lost after further adjustment for cardiovascular risk factors including age, hypertension, hypercholesterolaemia, diabetes mellitus, family history of cardiovascular disease, and GFR CKD-EPI (Table 3; multivariable Model 2). No significant association of PYY with 3-P-MACE was found by multivariable Cox regression analyses (Table 3).

## 4. Discussion

In this study, we found PYY levels to be associated with indicators of cardiovascular risk as well as cardiovascular events and all-cause mortality in patients with acute myocardial infarction. This association was, however, lost in more complex statistical models. Therefore, elevated PYY levels seem to be indicative for patients with increased cardiovascular risk and impaired prognosis, which is primarily attributable to its association with different risk factors.

Consistent with others, we found PYY serum levels to increase with age and to be elevated in patients with diabetes, which has similarly been reported by some [20] although not all investigators [21]. In addition, arterial hypertension was more often present in patients with high PYY levels, which might be attributable to direct vasoconstrictive effects of the peptide resulting in increased blood pressure under experimental conditions [22,23]. Furthermore, more patients with impaired kidney function had elevated PYY levels in our study, which is consistent with earlier findings of elevated PYY levels in patients with terminal renal insufficiency [24]. In addition, PYY correlated with NT-proBNP as an indicator of heart failure, recapitulating earlier findings of elevated PYY levels in patients with advanced heart failure and cardiac cachexia [25]. Moreover, PYY correlated with hs-CRP as a parameter of inflammation in our study. This might indicate an inflammatory regulation of PYY secretion, which has been reported for other gastrointestinal hormones including GLP-1, or result from a chronic inflammatory milieu created by the accumulation of different cardiovascular risk factors [26,27]. Finally, higher PYY levels were found in patients taking blood pressure-, glucose-, and lipid-lowering medication in addition to antiplatelet therapy. This might be indicative of a more severe state of disease in patients with high PYY levels, although drug-related effects on PYY secretion and/or catabolism cannot be excluded. Consequently, the ACE inhibitor captopril has been reported to inhibit PYY degradation [28]; others, however, found no effect of ACE inhibition on PYY breakdown [29]. Moreover, Metformin has been reported to stimulate PYY secretion [30].

Secretion of PYY happens in response to nutritional stimuli from enteroendocrine L-cells located in the distal gut. The same cell population produces GLP-1 in response to nutritional but also inflammatory stimuli, leading to elevated GLP-1 levels in patients with acute myocardial infarction or sepsis [4,31]. Importantly, GLP-1 was found to be an excellent and independent predictor for future cardiovascular events or mortality in both groups of patients, demonstrating a relevant interaction between the cardiovascular system and the gut [5,32]. Similar predictive power was not detected for PYY as an additional gut-derived peptide in this study. Nevertheless, PYY levels were associated with cardiovascular events and mortality by univariable analysis. This underlines a relevant crosstalk between the cardiovascular and intestinal system. Given the therapeutic potential of GLP-1 receptor agonists, including the reduction of cardiovascular events and cardiovascular mortality in high-risk patients with diabetes [6,7,8,9], it seems promising to further explore the cardiovascular function of other gut-derived peptides. These have so far mostly been studied as regulators of energy metabolism and gut motility [33]. PYY exists as a full-length PYY(1–36) peptide that binds to the neuropeptide Y1 and Y2 receptors and the PYY(3–36) peptide, which is derived from PYY(1–36) via processing by DPP-IV binding to the neuropeptide Y2 receptors [34]. Rise in PYY levels has been suspected to be the driving factor for early metabolic improvement following bariatric surgery [26]. Importantly, application of PYY or activation of its receptors leads to reduced appetite in obese individuals and promotes loss of body weight [17]. As a relevant limitation of this study, we did not assess body weight in our cohort and are therefore unable to report associations between PYY and Body-Mass-Index. Cardiovascular relevance of PYY was suggested by Y1- and Y2-receptor signaling known to modulate cardiac contractility [35]. Y1-receptor activation provided inotropic effects in isolated rat cardiomyocytes [36], and downregulation of Y1-receptor expression was found in rodent heart failure models and cardiac biopsies of patients with heart failure [37,38]. Further, Y1-receptor signaling was found to increase microvascular constriction in response to acute myocardial infarction [39]. In addition, upregulation of Y1 and Y2 receptors was found in atherosclerotic lesions with localization to smooth muscle cells, macrophages, and endothelial cells [40]. Vascular effects combined vasoconstriction in response to Y1-receptor signaling [41] and angiogenesis in response to Y2-receptor signaling [42]. Nonetheless, the relevance of PYY in the cardiovascular system remains largely unexplored and will require further investigation. Importantly, as total PYY serum levels were assessed in this study—which does not discriminate between PYY(1–36) and PYY(3–36)—this does not allow speculation about specific Y1- and/or Y2-receptor activation.

In conclusion, we found PYY to be associated with various cardiovascular risk factors in patients with acute myocardial infarction. We also found an association between PYY and cardiovascular events as well as all-cause mortality in this population. However, this association lost its significance after adjustment for further confounders. 

## Figures and Tables

**Table 1:** (**a**) Baseline characteristics. (**b**) Baseline medication.

**(a)**					
		**Tertiles of Peptide YY (PYY) (pg/mL)**			
**Characteristics**	**All Patients** **(*n* = 918)**	**≤114** **(*n* = 278)**	**114 to 168** **(*n* = 277)**	**>168** **(*n* = 279)**	***p*-Value ^a^**
Demographics					
Age, years	66.9 ± 12.7	64.3 ± 12.5	67.2 ± 12.8	69.3 ± 11.8	<0.0001
Sex (male)	672 (73.2%)	203 (73%)	212 (76.5%)	198 (71%)	0.5819
Cardiovascular risk factors					
Smoker	498 (59.4%)	159 (60.9%)	156 (61.4%)	136 (54.6%)	0.1517
Hypertension	664 (75.4%)	188 (71.2%)	193 (72.6%)	223 (83.2%)	0.0012
Hypercholesterolemia	452 (58.2%)	134 (57%)	130 (55.3%)	150 (62.8%)	0.2029
Diabetes mellitus	223 (25.4%)	49 (18.9%)	55 (20.5%)	94 (35.3%)	<0.0001
Systolic blood pressure (mmHg)	143.8 ± 23.2	145.8 ± 24.4	143.6 ± 22.8	141.8 ± 22.2	0.8464
Kidney disease	101 (11.2%)	17 (6.1%)	25 (9.1%)	48 (17.2%)	<0.0001
Liver disease	17 (1.9%)	4 (1.4%)	4 (1.5%)	9 (3.2%)	0.1356
COPD	76 (8.4%)	21 (7.6%)	17 (6.2%)	31 (11.1%)	0.1274
Atrial fibrillation	74 (8.2%)	17 (6.1%)	22 (8%)	30 (10.8%)	0.0471
GRACE score	149.1 ± 31.2	144.8 ± 28.2	150.3 ± 32.4	151.8 ± 32.1	0.0136
GRACE category					
Low, ≤108	67 (7.3%)	21 (7.6%)	22 (7.9%)	18 (6.5%)	
Medium, 108–140	314 (34.2%)	108 (38.8%)	86 (31%)	91 (32.6%)	
High, >140	537 (58.5%)	149 (53.6%)	169 (61%)	170 (60.9%)	
Previous cardiovascular disease					
Family history of CVD	305 (40.2%)	95 (39.8%)	100 (43.7%)	82 (37.1%)	0.5835
Myocardial infarction	218 (24.9%)	60 (22.2%)	66 (25.2%)	77 (28.8%)	0.0783
PTCA	244 (27.9%)	72 (26.8%)	70 (26.8%)	84 (31.9%)	0.1879
CABG	92 (10.4%)	28 (10.3%)	24 (9.1%)	33 (12.3%)	0.4658
Myocardial infarction subtype					
NSTEMI	597 (65.0%)	190 (68.3%)	174 (62.8%)	189 (67.7%)	0.8815
STEMI	321 (35.0%)	88 (31.7%)	103 (37.2%)	90 (32.3%)	
Risk markers at baseline					
hs-Troponin T (ng/mL)	146.3 (46.45–492.6)	137.2 (48.1–410.8)	176.5 (42.5–525.2)	126.5 (44.3–551.8)	0.9382
NT-proBNP (pg/mL)	663.6 (184.5–2271)	554 (168.6–1476.5)	639 (163.8–2222)	924.3 (261.4–3438)	<0.0001
hs-CRP (mg/L)	4.28 (1.70–15.26)	3.4 (1.7–10.5)	4.1 (1.6–16.6)	5.4 (1.8–20.6)	0.0061
Serum creatine kinase (U/L)	181 (107–398)	210 (123–501)	180 (105–391)	159.5 (94–298.5)	0.0002
Glucose (mg/dL)	129 (108–158)	125 (107–145)	124 (106–152)	137 (116–187)	<0.0001
GFR CKD-EPI (mL/min/1.73 m²)	75.8 ± 24.6	83.6 ± 19.7	78.3 ± 22.7	66.4 ± 26.1	<0.0001
**(b)**					
		**Tertiles of PYY (pg/mL)**			
**Premedication**	**All Patients** **(*n* = 697) ^b^**	**≤114** **(*n* = 222) ^b^**	**114 to 168** **(*n* = 204) ^b^**	**>168** **(*n* = 210) ^b^**	***p*-Value ^c^**
ACEi/ARB	333 (49.1%)	94 (43.3%)	94 (47.7%)	114 (55.9%)	0.0102
MRA	26 (3.9%)	8 (3.7%)	8 (4.1%)	8 (4%)	0.8826
Calcium Channel Blocker	121 (17.9%)	30 (13.8%)	36 (18.2%)	42 (20.7%)	0.0633
Beta blocker	290 (42.8%)	84 (38.9%)	80 (40.6%)	100 (48.8%)	0.0414
Antiplatelet therapy	277 (39.9%)	80 (36%)	72 (35.5%)	105 (50%)	0.0034
Phenprocoumon/Warfarin	46 (6.7%)	15 (6.8%)	12 (5.9%)	16 (7.8%)	0.6883
Statin	220 (32.0%)	60 (27.3%)	58 (28.9%)	85 (41.3%)	0.0022
Diuretic	209 (30.8%)	45 (20.6%)	60 (30.5%)	89 (43.6%)	<0.0001
Antidiabetic premedication					
Metformin	66 (9.6%)	14 (6.4%)	17 (8.4%)	27 (13.2%)	0.0160
Sulfonlyurea/Glinides	51 (7.4%)	10 (4.6%)	11 (5.5 %)	24 (11.7%)	0.0046
Insulin	56 (8.1%)	14 (6.3%)	5 (2.5%)	31 (15%)	0.0011

Continuous variables are expressed as mean ± SD or median (Q1–Q3) in case of skewed data. Categorical variables are shown as absolute and relative frequencies. ^a^ *p*-value of the test that Spearman’s rank correlation coefficient ρ ≠ 0 in the case of continuous characteristics or *p*-value of the Cochran–Armitage test in the case of nominal characteristics. Data are shown as absolute and relative frequencies. ^b^ No information on medication for 221 patients. ^c^ *p*-value of the Cochrane-Armitage test. ACEi = ACE inhibitor; ARB = Angiotensin II receptor blocker; CABG = Coronary artery bypass grafting; COPD = Chronic obstructive pulmonary disease; CVD = Cardiovascular disease; GFR CKD-EPI = Glomerular filtration rate (Chronic kidney disease epidemiology collaboration); GRACE = Global Registry of Acute Coronary Events; hs-CRP = high sensitivity C-reactive protein; hs-Troponin T = high sensitivity Troponin T; MRA = Mineralocorticoid receptor antagonist; NSTEMI = Non-ST-Elevation Myocardial Infarction; NT-proBNP = N-terminal prohormone of natriuretic brain peptide; PTCA = Percutaneous transluminal coronary angioplasty.

**Table 2:** Univariable Cox regression for log (PYY).

Survival Outcome	No. Events	Estimated Hazard Ratio (95% CI)	*p*-Value
Combined triple endpoint	56	1.7 (1, 2.97)	0.0495
All-cause mortality	60	2.69 (1.61, 4.47)	0.0001
Cardiovascular mortality	27	1.38 (0.63, 3.01)	0.4172
Nonfatal myocardial infarction	26	2.54 (1.12, 5.76)	0.0259
Nonfatal stroke	3	0.84 (0.08, 8.9)	0.8871
Rehospitalization	202	1.24 (0.94, 1.65)	0.1312
Coronary reintervention	83	0.87 (0.56, 1.36)	0.5389

**Table 3:** Multivariable Cox regression for log (PYY).

	Model 1		Model 2	
Survival Outcome	Estimated Hazard Ratio (95% CI)	*p*-Value	Estimated Hazard Ratio (95% CI)	*p*-Value
Combined triple Endpoint	1.52 (0.87, 2.65)	0.1402	0.66 (0.32, 1.35)	0.2517
All-cause mortality	2.26 (1.33, 3.83)	0.0024	1.06 (0.52, 2.18)	0.8687

Model 1 was adjusted for age and sex. Model 2 was adjusted for age, hypertension, hypercholesterolemia, diabetes mellitus, family history of cardiovascular disease and kidney function (GFR CKD-EPI).
